# Redox-Regulating Propolis- and Honey-Containing Hydrogels in Osteoarthritis

**DOI:** 10.3390/antiox15091202

**Published:** 2026-09-19

**Authors:** Jurate Zarauske, Greta Kaspute, Tatjana Ivaskiene

**Affiliations:** Department of Personalized Medicine, State Research Institute Centre for Innovative Medicine, LT-08406 Vilnius, Lithuania; jurate.zarauske@imcentras.lt (J.Z.); tatjana.ivaskiene@imcentras.lt (T.I.)

**Keywords:** redox regulation, inflammation, osteoarthritis, propolis, honey, hydrogels

## Abstract

Oxidative stress and persistent inflammation are central mechanisms in osteoarthritis (OA), contributing to chondrocyte dysfunction, mitochondrial impairment, extracellular matrix degradation, pain-related signaling, and progressive joint tissue remodeling. Reactive oxygen species (ROS) act not only as damaging agents but also as regulators of redox-sensitive inflammatory pathways, including NF-κB, MAPK, and Nrf2-related antioxidant responses. Accordingly, localized strategies capable of modulating the oxidative–inflammatory microenvironment may offer therapeutic advantages over non-targeted antioxidant approaches. This review summarizes current evidence on natural hydrogel-based systems containing propolis and honey as potential redox-modulating and anti-inflammatory platforms for OA-related tissue degeneration. Propolis and honey are bee-derived bioactive products rich in polyphenols and other redox-active compounds with antioxidant, anti-inflammatory, antimicrobial, and tissue-protective properties. Their incorporation into hydrogel matrices may support sustained local delivery, improved tissue retention, and stimulus-responsive release within inflamed or oxidatively stressed tissues. Particular attention is given to mechanisms involving ROS regulation, suppression of inflammatory mediators, activation of endogenous antioxidant defenses, and protection of cartilage extracellular matrix. Current formulation evidence is also critically interpreted according to its disease specificity, distinguishing direct OA/cartilage-related evidence from indirect wound healing and tissue repair data. The review also addresses intra-articular biomechanical requirements, enzyme-responsive degradation, immunological safety, formulation standardization, and translational barriers.

## 1. Introduction

Redox balance is a fundamental determinant of cellular function, regulating metabolism, intracellular signaling, immune responses, and tissue repair. Under physiological conditions, reactive oxygen species (ROS) act as tightly controlled signaling molecules that modulate cellular adaptation and homeostasis [1,2,3]. However, excessive ROS accumulation or impaired antioxidant defenses leads to oxidative stress, a condition associated with chronic inflammation, tissue degeneration, and disease progression [4,5,6].

Oxidative stress is increasingly recognized as a key contributor to degenerative joint diseases such as osteoarthritis (OA). In OA, disrupted redox homeostasis promotes chondrocyte dysfunction, mitochondrial impairment, extracellular matrix degradation, and persistent low-grade inflammation within the joint microenvironment [7,8,9,10,11]. Elevated ROS levels activate catabolic and redox-sensitive inflammatory pathways, increase the expression of matrix-degrading enzymes, and disturb cartilage homeostasis, thereby contributing to progressive joint degeneration [12,13,14,15,16].

Therapeutic strategies aimed at restoring oxidative homeostasis have therefore gained considerable attention. However, conventional systemic antioxidant supplementation has produced inconsistent effects, largely due to limited bioavailability, rapid clearance, and insufficient accumulation in poorly vascularized tissues such as articular cartilage [4,17,18]. These limitations have encouraged the development of localized and controlled delivery systems capable of modulating oxidative and inflammatory processes directly within the affected tissue microenvironment [17,18,19,20].

Hydrogels have emerged as versatile biomaterial platforms due to their high-water content, biocompatibility, tunable physicochemical properties, and ability to support sustained local delivery [19,21,22,23]. In OA-related applications, hydrogel systems are relevant because they can mimic aspects of the hydrated cartilage extracellular matrix, support cell viability, prolong bioactive compound retention, and contribute to tissue repair [18,19,20,24,25]. Natural hydrogels incorporating bioactive compounds may further combine structural functionality with intrinsic biological activity [21,26,27].

Among natural bioactive agents, bee-derived products such as propolis and honey have attracted interest because of their antioxidant, anti-inflammatory, antimicrobial, and tissue-protective properties [28,29,30,31,32,33]. These effects are largely related to their polyphenols and other redox-active constituents, which may modulate ROS production, endogenous antioxidant defenses, and redox-sensitive inflammatory signaling [34,35,36,37,38,39]. Their incorporation into hydrogel matrices is therefore evaluated in this review mainly through redox regulation, inflammation control, local retention, and controlled release.

The inclusion of wound healing and tissue repair studies in this review is based on shared redox-inflammatory mechanisms rather than on direct disease equivalence. Both wound repair and OA involve ROS accumulation, redox-sensitive inflammatory signaling, cytokine-mediated tissue responses, extracellular matrix remodeling, and activation of endogenous antioxidant defenses. However, skin and wound tissues are vascularized, externally accessible, and regenerative, whereas the osteoarthritic joint is characterized by avascular cartilage, synovial inflammation, mechanical loading, intra-articular clearance, and limited intrinsic cartilage repair. Therefore, wound-derived hydrogel studies are cited as mechanistic and formulation-level evidence, not as direct proof of OA efficacy. This distinction reframes the novelty of the review around the translational barriers and OA-specific microenvironmental challenges that propolis- and honey-containing hydro-gels must overcome before intra-articular application can be considered.

This review provides an integrative analysis of redox regulation in natural hydrogel-based systems containing propolis and honey, with emphasis on their potential relevance to OA pathogenesis and therapeutic modulation [40,41,42,43,44,45]. Attention is given to mechanisms involving oxidative stress, inflammation, cartilage matrix degradation, localized delivery, formulation challenges, disease-specific evidence gaps, biomechanical requirements, intra-articular safety, enzyme-responsive degradation, and translational requirements for OA validation [20,46,47,48,49,50,51].

## 2. Materials and Methods

This review was designed as an integrative narrative literature review focused on redox regulation, OA, and natural hydrogel-based biomaterials incorporating bee-derived bioactive compounds, namely propolis and honey. The literature search was conducted using major scientific databases, including PubMed, Scopus and Web of Science. The search strategy combined keywords related to redox biology, OA, hydrogel biomaterials, and bee-derived compounds. The following search terms and their combinations were used: “redox homeostasis”, “oxidative stress”, “reactive oxygen species”, “ROS-responsive hydrogels”, “osteoarthritis”, “cartilage degradation”, “chondrocytes”, “propolis”, “honey”, “bee products”, “natural hydrogels”, “antioxidant hydrogels”, “stimuli-responsive hydrogels”, “controlled drug delivery”, and “tissue repair”. Because of the limited OA-specific evidence, wound healing, burn, diabetic wound, and tissue repair studies were included only when they provided mechanistic or formulation-level information relevant to redox regulation, inflammation, biomaterial performance, or controlled release.

Articles were selected based on their relevance to the molecular mechanisms of redox regulation, inflammatory signaling, oxidative stress in OA, hydrogel-based delivery systems, and the antioxidant or anti-inflammatory properties of propolis and honey. Priority was given to peer-reviewed original research articles, while recent review articles were included primarily to support mechanistic background and conceptual integration. Studies lacking mechanistic relevance, insufficient methodological detail, or direct association with redox modulation, OA pathology, hydrogel-based delivery systems, or bee-derived bioactive compounds were excluded. The literature search was conducted between January 2020 and March 2026.

The selected literature was analyzed thematically and grouped into the following main categories: redox regulation in inflammation and tissue repair, oxidative stress in OA, alternative delivery systems for natural bioactive compounds, natural hydrogels as redox-modulating biomaterials, mechanisms of action of propolis and honey, and hydrogel-based systems incorporating bee-derived compounds. Attention was given to studies describing molecular pathways relevant to oxidative stress and inflammation, including ROS regulation, nuclear factor kappa-light-chain-enhancer of activated B cells (NF-κB), mitogen-activated protein kinase (MAPK), nuclear factor erythroid 2-related factor 2/heme oxygenase-1 (Nrf2/HO-1) signaling, mitochondrial dysfunction, matrix metalloproteinase activity, and extracellular matrix degradation.

Data from the selected studies were synthesized qualitatively due to heterogeneity in experimental models, hydrogel compositions, bioactive compound sources, formulation strategies, and outcome measures. Therefore, no meta-analysis was performed. This review was designed as an integrative narrative synthesis aimed at providing a mechanistic overview of current evidence, identifying technological advances, and highlighting research gaps in the field. The literature search identified 715 records in PubMed, 824 in Scopus, and 687 in Web of Science; after duplicate removal, 1024 records were screened, 287 full-text articles were considered, and 127 studies were included in the final narrative synthesis.

## 3. Results

### 3.1. Redox Regulation in Inflammation and Osteoarthritis

Redox homeostasis is essential for maintaining cellular integrity and coordinating inflammatory responses. ROS are generated through multiple pathways, including mitochondrial respiration and enzymatic systems such as nicotinamide adenine dinucleotide phosphate (NADPH) oxidases. At controlled levels, ROS function as signaling molecules that regulate immune cell activation, cytokine production, and antimicrobial defense [1,2,3,6,52,53].

**Figure 1:**
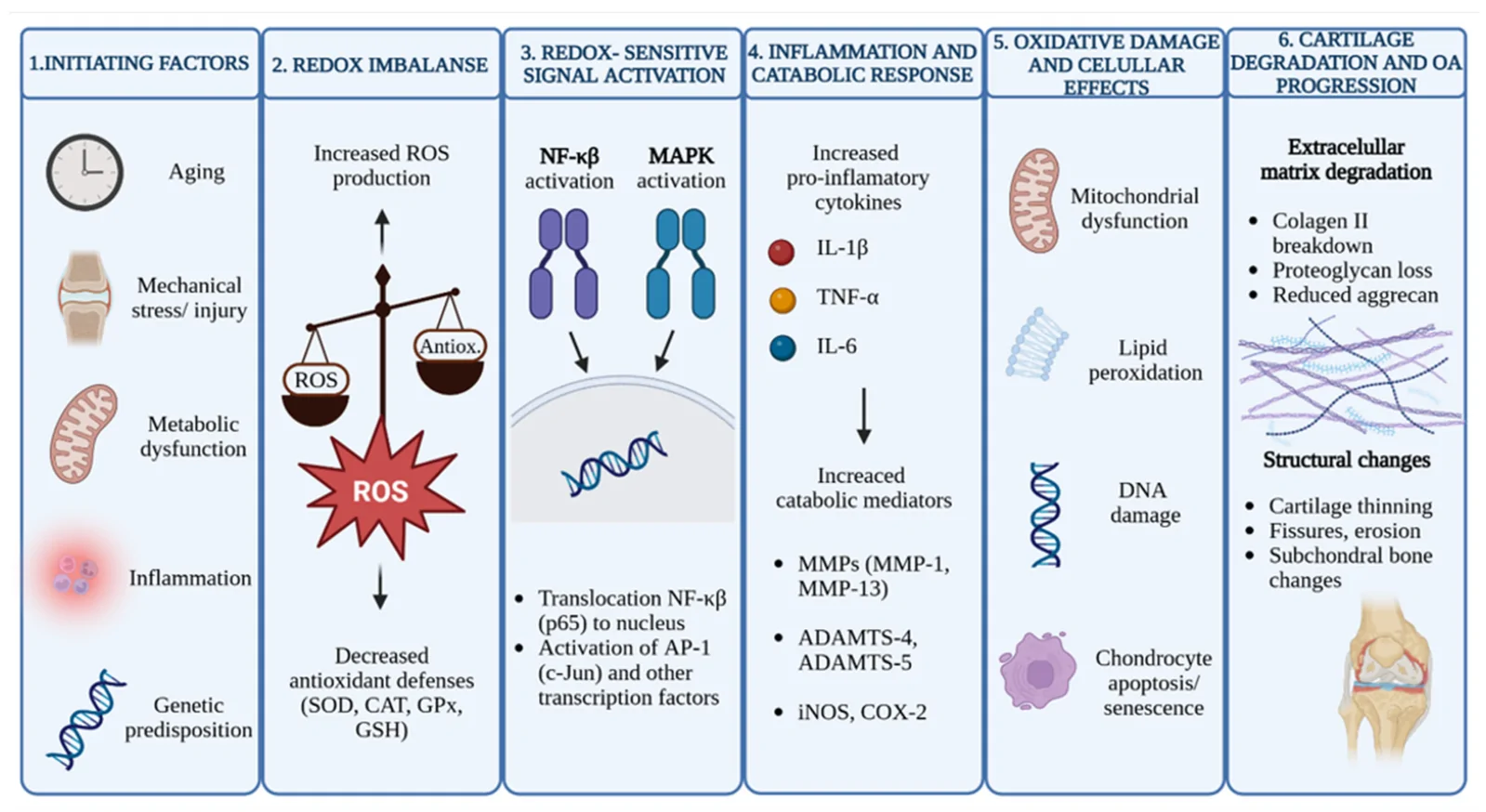
The redox-mediated pathogenesis and progression of OA. Created in BioRender. Ivaskiene, T. (2026) https://BioRender.com/zpyw534.

In OA tissues, however, dysregulated ROS production contributes to a pathological microenvironment characterized by oxidative stress, chronic inflammation, and progressive cartilage degradation [7,8,9,10,13]. Chondrocytes exposed to elevated ROS levels exhibit altered metabolic activity, increased apoptosis, and enhanced expression of catabolic mediators, further exacerbating joint degeneration [9,10,13,14,15,54]. The redox-mediated pathogenesis and progression of OA are summarized in Figure 1.

Excessive ROS production disrupts cellular homeostasis, leading to oxidative damage of lipids, proteins, and nucleic acids [2,3,6,53,55]. This imbalance amplifies inflammatory signaling through activation of redox-sensitive pathways, creating a self-sustaining cycle of oxidative stress and inflammation [7,11,15]. In OA, this cycle is further reinforced by mechanical stress and inflammatory mediators within the joint microenvironment, promoting disease progression [8,11,12,16].

Key regulators of redox signaling include transcription factors that respond to oxidative cues. Antioxidant defense systems are activated through adaptive mechanisms that restore redox balance, while pro-inflammatory pathways drive immune responses under stress conditions [6,14,15,56,57]. The interplay between these opposing processes determines the outcome of inflammation and tissue repair, and in OA, this balance is often shifted toward a catabolic and degenerative state [3,6,10,11,15,58].

Targeting redox imbalance at the site of injury or inflammation represents a promising therapeutic strategy when combined with biomaterials capable of controlled delivery and sustained activity. In OA treatment, such approaches may enable localized modulation of oxidative stress within the joint, potentially slowing cartilage degradation and improving tissue repair outcomes [17,18,19,20,24,59,60,61].

### 3.2. Alternative Delivery Systems and the Rationale for Hydrogel-Based Approaches

A broad range of alternative delivery systems has been investigated for natural bioactive compounds such as propolis and honey. These include lipid-based carriers, liposomes, nanoemulsions, polymeric nanoparticles, and conventional topical formulations, all of which may enhance encapsulation efficiency, solubility, physicochemical stability, bioavailability, and short-term availability of redox-active compounds [34,35,36,37,38,48,60,61,62]. However, carrier-only approaches may still be limited by insufficient retention at the target site, uncontrolled release kinetics, and the need for secondary matrices to improve local tissue interaction [46,47,48,60,61].

Hydrogel-based systems offer a more integrative platform than carrier-only approaches because they combine controlled delivery with local tissue retention and structural support. Unlike conventional nanocarriers, hydrogels can function as three-dimensional biomaterial matrices that regulate bioactive compound release while interacting with the surrounding tissue microenvironment [19,20,21,22,49]. This is relevant for redox-active compounds such as propolis- and honey-derived constituents, whose therapeutic activity depends not only on antioxidant capacity but also on sustained presence at the target site. When combined with ROS-responsive components, hydrogel systems may further enable stimulus-triggered release under oxidative conditions, supporting localized redox modulation in OA-related microenvironments [60,61,63,64].

In this review, advanced delivery technologies are considered primarily according to their ability to improve local retention, controlled release, and redox-responsive behavior in OA-relevant microenvironments, rather than as independent therapeutic platforms.

Beyond delivery, emerging sensing and molecular recognition technologies may provide additional tools for monitoring, standardizing, and regulating natural bioactive compounds. Molecularly imprinted polymers are designed with template-specific recognition sites that allow selective binding of target molecules and may therefore be useful for detecting or capturing selected phytochemicals [65,66,67,68]. Although current studies have mainly focused on model naturally derived compounds such as geraniol, the same molecular recognition principles could potentially be adapted to selected propolis- or honey-derived constituents. Such systems may support chemical standardization, selective compound handling, and, in future designs, controlled release within biomaterial platforms.

Overall, the integration of nanocarriers, hydrogel matrices, ROS-responsive components, and molecular recognition systems may support the development of multifunctional redox-modulating platforms. For propolis- and honey-based biomaterials, this approach may help overcome current limitations related to poor stability, compositional variability, limited tissue retention, and insufficient control over release behavior. However, the therapeutic relevance of these advanced systems, especially in osteoarthritis, still requires disease-specific validation, standardized formulation strategies, and direct evaluation in OA-relevant in vitro and in vivo models.

### 3.3. Natural Hydrogels as Redox-Modulating Biomaterials

Natural hydrogels have gained significant attention as platforms for delivering bioactive compounds due to their biocompatibility, biodegradability, and ability to mimic the extracellular matrix [21,22]. These materials can be engineered to provide controlled release of therapeutic agents, making them suitable for localized modulation of oxidative stress. In OA, such localized delivery is especially advantageous, as it enables targeted modulation of oxidative stress within the joint microenvironment, where systemic therapies often show limited efficacy [17,18,19,20,22,23,25]. In addition to their biological compatibility, hydrogels exhibit a range of tunable physicochemical properties that are critical for their performance in biomedical applications. These include high water content, viscoelastic behavior, and adjustable crosslinking density, all of which influence their mechanical strength, swelling capacity, and diffusion characteristics [19,20,21,23].

In redox regulation, hydrogels serve not only as carriers but also as functional systems that interact with the cellular microenvironment. Their porous structure facilitates diffusion of oxygen and nutrients, while their composition can be tailored to incorporate antioxidant molecules or redox-responsive components [20,24,60,61]. Importantly, parameters such as pore size, network architecture, and degradation rate can be precisely controlled, enabling modulation of mass transport and temporal release profiles of embedded bioactive agents [21,26,46,49,69,70].

Natural polymers such as chitosan, alginate, gelatin, and hyaluronic acid are commonly used to fabricate hydrogels with desirable mechanical and biological properties. These polymers differ substantially in their chemical structure, charge characteristics, and gelation mechanisms, which in turn determine their functional behavior in biomedical applications [21,26,27,71,72,73]. Chitosan, a cationic polysaccharide derived from chitin, is characterized by its positive charge, enabling electrostatic interactions with negatively charged biomolecules and cell membranes, as well as intrinsic antimicrobial activity [71,73,74,75]. Alginate, in contrast, is an anionic polysaccharide that forms hydrogels through ionic crosslinking with divalent cations such as calcium, resulting in relatively stable but mechanically brittle networks with limited cell adhesion capacity [27,76]. Gelatin, a denatured form of collagen, retains bioactive peptide sequences (e.g., Arginine–Glycine–Aspartic acid (RGD) motifs) that promote cell adhesion and proliferation, but its thermal sensitivity can limit structural stability under physiological conditions [72,77]. Hyaluronic acid, a naturally occurring glycosaminoglycan, is highly hydrophilic and plays a key role in tissue hydration and extracellular matrix organization, although it often requires chemical modification to achieve sufficient mechanical strength and controlled degradation [26].

These intrinsic differences influence not only gel formation and stability but also critical physicochemical properties such as swelling behavior, degradation kinetics, and responsiveness to environmental stimuli [21,26,27,73]. For instance, chitosan-based hydrogels are often pH-responsive due to protonation of amino groups, whereas alginate systems are more sensitive to ionic environments [27,73,74]. Gelatin-based hydrogels can exhibit temperature-dependent sol–gel transitions, while hyaluronic acid-based systems can be engineered for enzyme-responsive degradation and improved mechanical properties [26,72,73]. When combined with bioactive substances, these materials can differentially modulate antioxidant activity, inflammatory responses, and cellular behavior depending on their interaction with the surrounding microenvironment [46,48,49,50,69,70]. Consequently, the selection and design of polymer systems play a pivotal role in tailoring hydrogel functionality for specific therapeutic applications, including the controlled delivery of redox-modulating compounds [21,24,60,61,78].

For intra-articular OA application, hydrogel performance must be evaluated beyond biochemical antioxidant activity. Candidate systems should exhibit shear-thinning injectability, rapid structural recovery after injection, appropriate viscoelasticity for mixing with synovial fluid, resistance to repeated mechanical loading, and adequate lubrication at the cartilage surface. Chitosan- and alginate-based hydrogels may require additional crosslinking or blending to improve stability under high shear stress, whereas hyaluronic acid-based systems are intrinsically relevant to joint lubrication but often require modification to prolong residence time. Gelatin-based systems may provide cell-interactive motifs but can be susceptible to enzymatic degradation. Incorporation of viscous honey or resinous propolis may alter viscosity, swelling, diffusion, frictional behavior, and therefore tribological performance; these properties should be quantified using rheological and tribological assays under joint-relevant loading conditions.

Importantly, the integration of natural antioxidants into hydrogel systems enables sustained and localized redox modulation, overcoming limitations associated with systemic administration. In OA conditions, this approach offers the potential to slow disease progression by mitigating oxidative stress–driven cartilage degeneration and improving the local biochemical environment [20,22,23,24,25,60,61]. Moreover, the ability to design stimuli-responsive hydrogels—capable of undergoing structural or functional changes in response to oxidative conditions—offers additional opportunities for dynamic and site-specific therapeutic intervention [24,60,61,63,64,78].

### 3.4. Propolis and Honey in Redox Regulation: Mechanisms and Complementary Potential

Propolis and honey are natural bee-derived substances with complex and complementary biochemical compositions that underpin their potent roles in redox regulation. Propolis, a resinous material collected from plant exudates, is rich in polyphenols, flavonoids, and phenolic acids, which confer strong antioxidant and anti-inflammatory properties [30,31,79,80]. At the molecular level, propolis modulates intracellular oxidative balance by regulating ROS levels and enhancing endogenous antioxidant defenses through activation of adaptive cellular pathways [28,30,31,79]. In addition, it suppresses redox-sensitive inflammatory signaling, thereby attenuating the amplification of oxidative stress and inflammation [28,31,81]. In OA joints, these effects are highly relevant, as propolis has been shown to inhibit redox-sensitive pathways such as NF-κB and MAPK, reducing the expression of pro-inflammatory cytokines and matrix-degrading enzymes and support cartilage homeostasis [40,41,82]. Propolis also influences mitochondrial function and cellular stress responses, including autophagy, contributing to the maintenance of cellular homeostasis [28,40]. Notably, its capacity to exhibit both antioxidant and context-dependent pro-oxidant activity further expands its therapeutic versatility [30,79].

Honey, in contrast, represents a multifaceted redox-modulating system composed of sugars, enzymes, vitamins, and a diverse range of polyphenolic compounds [32,33,83,84,85,86,87,88]. Its antioxidant activity is largely attributed to its phenolic profile, which varies depending on botanical origin and directly contributes to its ability to scavenge ROS and regulate redox-sensitive signaling pathways [32,33,83,84,86,87,88]. Beyond its antioxidant effects, honey plays a significant role in modulating immune responses and promoting tissue repair, as evidenced by its widespread application in wound healing [42,51,85,86,89,90]. Within OA conditions, honey may contribute to the reduction in oxidative stress in chondrocytes, support extracellular matrix preservation, and enhance cellular viability under inflammatory stress [29,91]. Its antimicrobial properties, combined with its capacity to reduce oxidative stress and support cellular regeneration, further enhance its therapeutic relevance [33,42,84,87,90].

The combination of propolis and honey may provide complementary redox-modulating effects because their biochemical profiles act through partly distinct mechanisms [43,44,45,92]. While propolis primarily targets intracellular signaling pathways and enhances antioxidant defenses, honey contributes strong radical-scavenging activity and supports tissue repair [30,31,32,79,80,86,90]. In OA joints, such complementary activity may support simultaneous suppression of inflammatory signaling and protection of cartilage from oxidative damage [29,40,41,82,91]. However, true synergy requires direct experimental validation using propolis-alone, honey-alone, combined formulations, blank hydrogels, and comparator groups. Table 1 summarizes the comparative redox-modulating mechanisms of propolis and honey. When integrated into hydrogel-based delivery systems, their combined activity may support sustained release, prolonged antioxidant activity, and enhanced interaction with the local tissue microenvironment [46,47,48,49,50,51,92,93,94]. Such an approach is advantageous in applications requiring continuous modulation of oxidative stress, including wound healing and chronic inflammatory conditions [42,51,92,95,96].

### 3.5. Natural Hydrogels Incorporating Propolis and Honey

The incorporation of bioactive natural compounds such as propolis and honey into hydrogel systems has led to the emergence of multifunctional biomaterials capable of simultaneously providing structural support and biological activity [46,48]. These hybrid systems represent a convergence of material science and redox biology, enabling localized and sustained modulation of oxidative stress within damaged or inflamed tissues [49,51,93,94].

From a physicochemical perspective, hydrogels serve as three-dimensional polymeric networks capable of retaining large amounts of water while maintaining mechanical integrity [49]. When functionalized with propolis and honey, these networks become active redox-modulating environments rather than passive delivery matrices [95,97]. The interaction between the hydrogel scaffold and embedded bioactive compounds is critical, as it influences release kinetics, stability, and biological efficacy [46,47,51,98].

**Figure 2:**
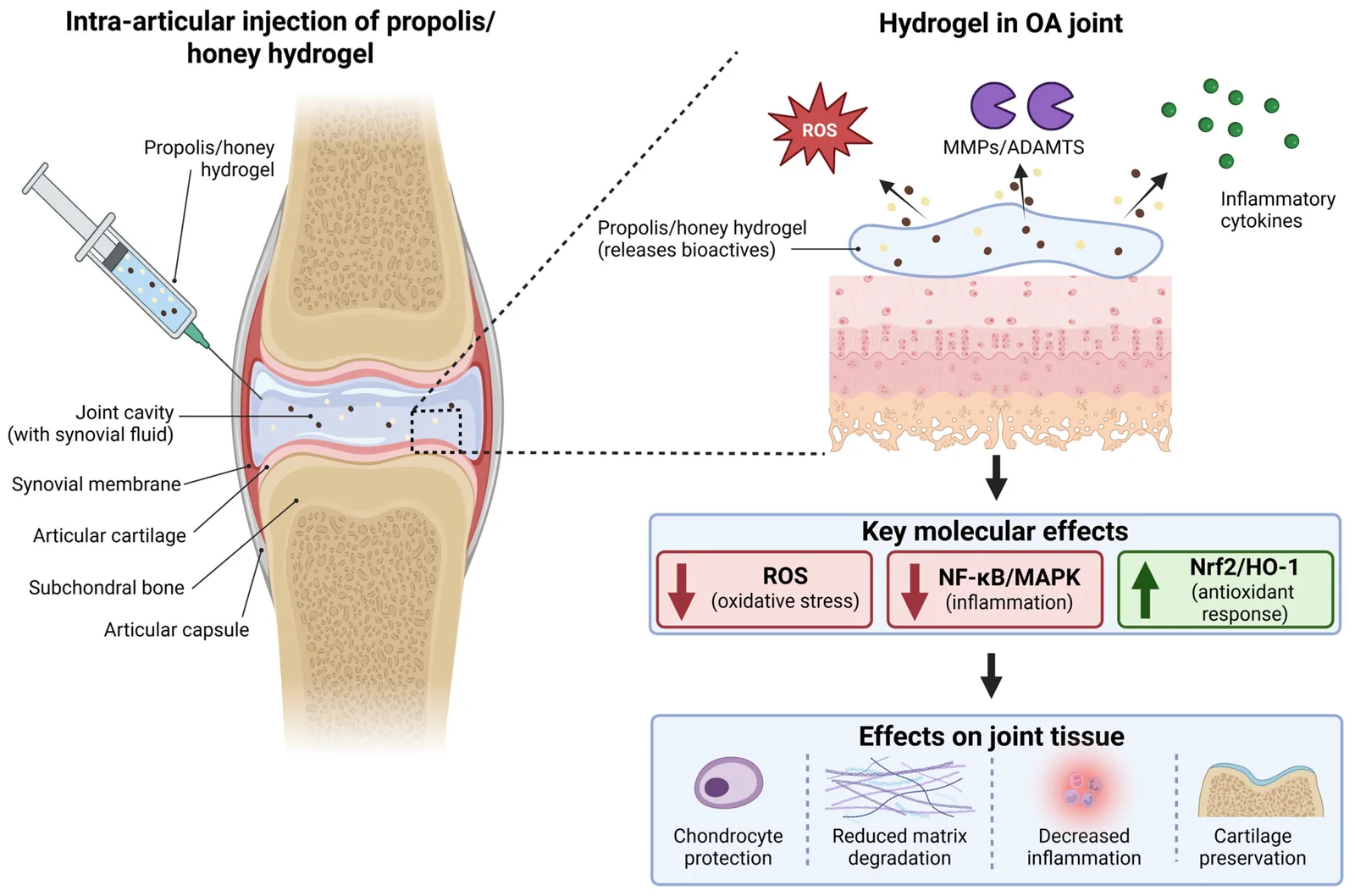
Intra-articular delivery and redox-responsive action of propolis–honey hydrogel in the osteoarthritic joint. Created in BioRender. Ivaskiene, T. (2026) https://BioRender.com/nqkja8x.

One of the key advantages of incorporating propolis and honey into hydrogels lies in their ability to provide controlled and sustained release of antioxidant compounds. Unlike free-form administration, where rapid degradation and systemic dilution limit efficacy, hydrogel systems allow gradual diffusion of polyphenols and flavonoids into the surrounding tissue [46,47,48,51,95]. This sustained delivery maintains therapeutic concentrations over extended periods, which is important in chronic inflammatory environments characterized by persistent oxidative stress [24,60,61,63,64]. Figure 2 illustrates the integration of propolis and honey into hydrogel systems.

The choice of polymer matrix plays a decisive role in determining the performance of these systems. Natural polymers such as chitosan, alginate, gelatin, and hyaluronic acid are widely used due to their biocompatibility and inherent bioactivity [21,26,27,72,73]. Chitosan-based hydrogels, for instance, offer intrinsic antimicrobial and wound-healing properties, which can complement the antioxidant effects of propolis and honey [69,71,73,74,75,96,97,99,100,101,102,103]. Alginate and gelatin systems provide tunable mechanical strength and porosity, allowing optimization of oxygen diffusion and nutrient transport, both of which are critical for tissue regeneration and redox balance [27,70,72,76,77,99,101,104,105,106].

In addition to passive release, advanced hydrogel systems can be engineered to exhibit stimuli-responsive behavior, enabling dynamic interaction with the oxidative microenvironment. Redox-responsive hydrogels are designed to undergo structural or chemical changes in response to variations in ROS levels [63]. For example, hydrogels containing redox-sensitive linkages can degrade in oxidative conditions, triggering the release of encapsulated antioxidants precisely at sites of elevated oxidative stress [64]. This targeted delivery approach enhances therapeutic efficiency while minimizing off-target effects [24,49,60,78].

In addition to ROS-responsive release, enzyme-responsive degradation is particularly relevant to OA. The osteoarthritic joint contains increased matrix-degrading activity, including MMPs and ADAMTS aggrecanases, which contribute to collagen and aggrecan breakdown. Natural hydrogel matrices such as gelatin, hyaluronic acid, and chitosan-based systems may be susceptible to enzymatic remodeling or can be engineered with enzyme-cleavable linkages. This feature could be leveraged for on-demand release of propolis- or honey-derived compounds in regions where catabolic enzyme activity and cartilage matrix degradation are elevated, provided that degradation kinetics are con-trolled and do not compromise joint residence time or safety.

The incorporation of propolis into hydrogels significantly enhances their antioxidant capacity through multiple mechanisms. Propolis-derived polyphenols not only scavenge ROS but also activate intracellular antioxidant pathways in surrounding cells, thereby amplifying the overall redox-modulating effect [28,30,31,79,80,107]. Moreover, propolis can interact with polymer chains and influence hydrogel matrix properties, potentially affecting crosslinking density, mechanical behavior, and release kinetics [46,49,69]. These interactions can improve the durability and functional lifespan of the hydrogel in physiological environments.

Similarly, honey plays a complementary role within hydrogel systems by providing immediate antioxidant activity and supporting tissue regeneration. Its high osmolarity and enzymatic activity contribute to antimicrobial effects, while its polyphenolic content enhances ROS scavenging [32,33,83,84,92,108,109]. In hydrogel formulations, honey improves hydration and creates a favorable microenvironment for cell proliferation and migration. Additionally, its viscosity and hygroscopic nature can influence the rheological properties of the hydrogel, contributing to improved adherence and coverage in wound applications [51,93,95,109,110].

The combination of propolis and honey within a single hydrogel system may provide complementary redox-regulating effects, but this should not be assumed without comparative validation. While propolis primarily modulates intracellular signaling and enhances endogenous antioxidant defenses, honey provides rapid ROS neutralization and supports extracellular matrix interactions [30,32,33,35,79,80,83,84,85,86,90]. This dual mechanism allows for both immediate and sustained control of oxidative stress [43,44,45,90,92,111]. Nevertheless, future studies should determine whether combined propolis-honey formulations produce effects beyond additivity on ROS levels, inflammatory mediators, matrix-degrading enzymes, and extracellular matrix preservation [43,44,45,90,92].

From a biological standpoint, propolis and honey-based hydrogels have demonstrated significant potential in promoting tissue repair. By reducing oxidative stress and inflammation, these systems create an environment conducive to cell survival, proliferation, and differentiation [46,47,48,49,50,51,62,96]. These wound healing studies are included because they inform shared redox-inflammatory mechanisms relevant to OA, including ROS accumulation, NF-κB/MAPK-related inflammatory signaling, Nrf2-linked antioxidant responses, inflammatory mediator production, MMP-associated matrix remodeling, and extracellular matrix repair processes. In wound healing applications, they have been associated with accelerated re-epithelialization, enhanced collagen deposition, improved antimicrobial protection, and reduced inflammatory burden [62,92,96,110,112,113]. However, these models differ from OA because skin and wound tissues are generally vascularized, externally accessible, and dominated by reparative remodeling, whereas OA involves avascular cartilage, synovial inflammation, mechanical loading, subchondral bone changes, and intra-articular clearance. Accordingly, wound-derived studies should be treated as indirect mechanistic or formulation-level evidence, while direct OA/cartilage relevance requires dedicated joint-specific validation. Table 2A,B summarize this distinction between indirect wound/soft-tissue evidence and cartilage/OA-relevant hydrogel evidence.

Note: Most formulations summarized in Table 2A should be interpreted as indirect mechanistic or formulation-level evidence for OA, because they were developed primarily for wound healing, burn, diabetic wound, or tissue repair applications. Table 2B summarizes the limited cartilage/OA-related hydrogel evidence and translational benchmark studies that define the disease-specific requirements for future propolis- and honey-containing systems.

Despite these promising features, several challenges must be addressed to fully realize the potential of these systems. Variability in the composition of propolis and honey can lead to significant inconsistencies in hydrogel performance, affecting not only antioxidant capacity but also release behavior and biological activity [95,96]. This variability arises from differences in botanical origin, geographic conditions, and processing methods, making standardization a critical prerequisite for reliable application [30,32,79,84]. Without rigorous standardization and precise characterization of bioactive components, it becomes difficult to ensure reproducibility, compare results across studies, or establish clear structure–function relationships [88]. Moreover, the lack of standardized profiles limits regulatory approval and clinical translation, as consistency, safety, and predictability are fundamental requirements in biomedical applications. Therefore, systematic approaches to raw material standardization, including chemical fingerprinting and quantification of key active compounds, are essential for advancing these systems beyond experimental settings [30,32,79,88].

Additionally, optimizing the balance between mechanical stability and release kinetics remains a critical design consideration. Overly dense networks may limit diffusion of bioactive compounds, whereas highly porous structures may compromise structural integrity and reduce retention at the target site [21,26,27,72,73]. Therefore, future hydrogel design should carefully integrate polymer selection, crosslinking strategy, pore architecture, degradation behavior, and bioactive compound loading to achieve controlled release, mechanical compatibility, and sustained redox-modulating activity [21,24,60,61,63,64,78].

## 4. Discussion

This review highlights propolis- and honey-containing hydrogels as localized platforms for modulating the oxidative–inflammatory microenvironment of OA. In OA, excessive ROS production, mitochondrial dysfunction, cytokine-driven inflammation, and extracellular matrix degradation are closely interconnected processes that promote chondrocyte dysfunction and progressive joint damage [7,8,11,15]. Therefore, therapeutic systems capable of regulating both oxidative stress and inflammatory signaling may offer advantages over non-targeted antioxidant approaches, especially in poorly vascularized tissues such as articular cartilage. In this context, redox modulation should be viewed not only as an antioxidant strategy but also as a means of influencing inflammation-associated signaling networks that sustain OA progression.

Hydrogel-based systems are relevant because they may improve local retention, sustained release, and spatial control of redox-active compounds. When combined with redox-responsive or nanoengineered components, these systems can potentially provide stimulus-sensitive release under oxidative conditions, linking compound delivery to local disease activity [24,60,61,63,64,78]. This is important in chronic inflammatory joint disease, where prolonged modulation of pathways such as NF-κB, MAPK, Nrf2/HO-1, cytokine production, MMP activity, ADAMTS expression, and cartilage matrix preservation may be required. Thus, the main relevance of hydrogel-based platforms in OA is their potential to maintain anti-inflammatory and redox-active compounds within the joint microenvironment for sufficient duration to affect disease-related signaling. However, this biochemical rationale must be integrated with the primary physical functions expected from intra-articular materials, including lubrication, load distribution, shear tolerance, and residence within synovial fluid.

Propolis and honey are pharmacologically relevant bee-derived products due to their polyphenols and other redox-active constituents. Propolis may primarily contribute through intracellular signaling modulation, suppression of redox-sensitive inflammatory pathways, and activation of endogenous antioxidant defenses [28,40,41,82]. Honey may provide complementary effects through ROS scavenging, antimicrobial activity, osmotic effects, and support of tissue repair processes [32,33,84,86]. However, propolis–honey synergy should be interpreted cautiously, as true synergistic effects require direct comparison of propolis alone, honey alone, combined formulations, blank hydrogels, and appropriate pharmacological comparators. Until such evidence is available, their combined effects should be described as complementary rather than synergistic.

The current evidence base remains limited for OA-specific application. Many available studies on propolis- or honey-containing hydrogels are derived from wound healing models, where oxidative stress and inflammation are relevant but not fully representative of the osteoarthritic joint. Their inclusion in this review is justified at the mechanistic level because wound repair and OA share several redox-inflammatory processes, including ROS accumulation, activation of NF-κB/MAPK signaling, Nrf2-related antioxidant responses, cytokine-driven tissue responses, MMP-associated extracellular matrix remodeling, and oxidative stress-related cell dysfunction. Accordingly, wound-derived studies can inform antioxidant capacity, inflammation modulation, biocompatibility, release behavior, and tissue-response profiles of propolis- and honey-containing hydrogel systems. However, they cannot be interpreted as direct evidence of OA therapeutic efficacy. The OA microenvironment differs substantially from wound tissue because it involves cartilage avascularity, synovial inflammation, altered mechanotransduction, subchondral bone remodeling, and pain-related inflammatory signaling. Therefore, extrapolation from wound healing models to OA should be presented as hypothesis-generating rather than confirmatory. Future studies should therefore use OA-relevant chondrocyte cultures, cartilage explants, synoviocyte cultures, co-culture systems, and in vivo models to evaluate inflammatory mediators, oxidative stress markers, cartilage degradation, pain-related outcomes, intra-articular retention, and release kinetics.

For intra-articular application, additional formulation requirements must be considered. Hydrogels intended for use in a high-load, dynamic joint environment should be evaluated for injectability, viscoelasticity, mechanical stability, degradation behavior, swelling, retention time, and release kinetics under inflammatory and ROS-rich conditions. These requirements differ from those of static wound dressings, where surface adherence and moisture retention are often the primary functional parameters. In OA, premature hydrogel degradation or rapid clearance from the joint cavity may limit therapeutic exposure, whereas excessive persistence or inappropriate stiffness may impair tissue compatibility. The effects of honey viscosity and propolis resinous components on friction, diffusion, hydrogel network density, and synovial-fluid interaction should therefore be assessed be-fore intra-articular translation. Accordingly, future studies should directly test whether propolis- and honey-containing hydrogels can maintain controlled release and anti-inflammatory activity under joint-relevant mechanical and biochemical conditions.

Several translational barriers also remain. The composition of propolis and honey varies according to botanical origin, geography, season, extraction method, and processing conditions, which may affect antioxidant capacity, anti-inflammatory activity, antimicrobial effects, and release behavior [30,32,79,88]. Standardized chemical fingerprinting, marker compound quantification, batch-to-batch quality control, sterility testing, allergenicity assessment, cytotoxicity evaluation, and comparison with established intra-articular therapies are required before these systems can be considered realistic therapeutic candidates for OA. In addition, dose–response relationships, degradation behavior under ROS-rich conditions, local immune compatibility, and potential allergenicity in synovial tissues should be established before clinical translation is considered.

A dedicated safety issue concerns the immunological profile of bee-derived products in the synovial cavity. Propolis contains sensitizing constituents, including caffeic acid and cinnamic acid derivatives, and topical propolis is associated with allergic contact dermatitis. Although this does not directly predict the response to intra-articular delivery, injection of allergenic compounds into synovial tissues could theoretically provoke synovitis or local immune activation. Mitigation strategies should include chemical profiling to identify allergenic markers, removal or reduction in sensitizing fractions, when possible, endotoxin and sterility testing, dose-escalation cytotoxicity studies, synoviocyte/macrophage inflammatory assays, and in vivo evaluation of synovial histopathology before therapeutic claims are made.

As a future perspective, MIPs and sensing-assisted systems may support chemical standardization and release monitoring of propolis- and honey-derived marker compounds, but they should not be presented as established OA-directed therapeutic components [65,68,122,123].

## 5. Conclusions

Propolis- and honey-containing natural hydrogels represent a biologically plausible approach for localized modulation of oxidative stress and inflammation in osteoarthritis. By combining the structural and delivery-related properties of hydrogel matrices with the intrinsic antioxidant, anti-inflammatory, antimicrobial, and tissue-protective activities of bee-derived bioactive compounds, these systems may provide a targeted strategy for influencing the oxidative–inflammatory processes involved in joint degeneration.

In OA, such platforms may be relevant for reducing ROS-driven inflammatory signaling, supporting endogenous antioxidant defenses, limiting chondrocyte dysfunction, and contributing to extracellular matrix preservation. Propolis and honey may act through complementary mechanisms: propolis mainly through modulation of redox-sensitive intracellular pathways, including NF-κB, MAPK, and Nrf2-related responses, and honey through ROS scavenging, antimicrobial activity, and support of tissue repair. However, their combined effects should be interpreted as complementary rather than definitively synergistic, until confirmed in direct comparative OA-specific studies.

Nanoengineered, redox-responsive, and sensing-assisted approaches may further improve the stability, bioavailability, local retention, monitoring, and controlled release of propolis- and honey-derived constituents. For OA, future systems should also be assessed for enzyme-responsive degradation, rheological behavior, tribological performance, intra-articular clearance, and local immunological safety. Nevertheless, important barriers remain, including compositional variability, lack of standardized chemical profiles, uncertain dose–response relationships, safety concerns, and limited validation in OA models. These limitations are relevant for inflammatory joint disease, where reproducible anti-inflammatory efficacy, local tissue compatibility, and long-term safety must be demonstrated.

Future research should therefore prioritize standardized formulations, marker compound characterization, cytotoxicity and sterility testing, release-profile analysis, and evaluation in chondrocyte cultures, synoviocyte cultures, cartilage explants, co-culture systems, and in vivo OA models. Because much current evidence derives from wound healing and tissue repair models, future studies should explicitly distinguish shared redox-inflammatory mechanisms from OA-specific therapeutic efficacy. Only through such disease-specific and inflammation-oriented validation can propolis- and honey-based hydrogels be considered realistic candidates for targeted anti-inflammatory and redox-modulating therapy in osteoarthritis.

## Figures and Tables

**Table 1:** Comparative redox-modulating mechanisms of propolis and honey.

Aspect	Propolis	Honey	Key Similarities/Differences
Primary composition	Rich in flavonoids, phenolic acids, terpenes	Composed of sugars, enzymes, and polyphenols	Both contain polyphenols, but propolis is more concentrated in bioactive compounds
ROS regulation	Modulates ROS production and intracellular levels	Primarily scavenges ROS directly	Propolis acts as a regulator; honey mainly as a neutralizer
Antioxidant mechanisms	Activates endogenous antioxidant enzymes (superoxide dismutase, catalase, and glutathione peroxidase)	Direct antioxidant activity with limited signaling effects	Propolis enhances intrinsic defense; honey provides immediate protection
Effect on signaling pathways	Strong modulation of Nrf2, NF-κB, MAPK pathways	Weaker modulation of signaling pathways	Propolis has a more pronounced intracellular regulatory role
Anti-inflammatory effects	Suppresses pro-inflammatory cytokines (tumor necrosis factor-alpha and interleukin-6 (TNF-α, IL-1β))	Reduces inflammation primarily via ROS reduction	Both are anti-inflammatory but act through different mechanisms
Mitochondrial function	Modulates mitochondrial stress and autophagy	Indirect effects on mitochondrial function	Propolis exerts deeper intracellular effects
Antimicrobial activity	Strong, broad-spectrum antimicrobial effects	Strong antimicrobial activity (hydrogen peroxide production, osmotic effects)	Both are effective but through distinct mechanisms
Bioavailability	Limited, dependent on formulation	Relatively high due to solubility	Honey is generally more bioavailable
Role in hydrogels	Enhances redox signaling and antioxidant defense	Improves biocompatibility and moisture retention	Complementary functions within hydrogel systems
Potential complementary effects	Enhances intracellular signaling modulation	Strengthens antioxidant and regenerative effects	Combination may enable multi-level redox regulation; true synergy requires comparative validation
Relevance in OA	Modulates redox-sensitive pathways (NF-κB, MAPK), reduces pro-inflammatory cytokines (IL-1β, TNF-α) and matrix-degrading enzymes, thereby limiting cartilage degradation	Scavenges ROS, supports chondrocyte viability, promotes extracellular matrix preservation and tissue repair under oxidative stress	Complementary actions may support simultaneous suppression of inflammation and protection of cartilage, but OA-specific validation remains limited

**Table 2:** (**A**) Propolis- and honey-containing hydrogel systems evaluated mainly in wound healing, burn, diabetic wound, or soft-tissue repair models. (**B**) Cartilage/OA-relevant hydrogel evidence and translational benchmarks for future propolis- and honey-containing intra-articular systems.

(**A**)					
**Bioactive Component**	**Hydrogel Composition**	**Experimental Model/Application**	**Key Outcomes**	**Biomarkers/Parameters Evaluated**	**Ref.**
Honey	Chitosan/gelatin/polyvinyl alcohol (PVA) + honey	In vitro and in vivo (rat model), wound healing	Enhanced healing, improved elasticity	Wound closure, histology, antibacterial activity	[95]
Honey	Chitosan/hyaluronic acid + honey	In vitro and in vivo (rat model), wound healing	Increased proliferation and adhesion, accelerated wound healing	Cell proliferation, swelling, porosity, wound healing	[100]
Honey	Alginate + anthocyanin + honey	In vitro	pH-responsive, antioxidant, antibacterial	Antioxidant activity, pH response, bacterial inhibition	[114]
Honey	Sericin + honey injectable hydrogel	In vitro and in vivo (mouse model), diabetic wound model	Accelerated healing, reduced oxidative stress	ROS levels, wound closure, 2,2-diphenyl-1-picrylhydrazyl (DPPH), antibacterial activity, wound healing	[113]
Kelulut honey	Gelatin/PVA + genipin + honey	In vitro biomaterial characterization	Good biocompatibility and stability	Physicochemical analysis, mechanical strength, cytotoxicity	[104]
Manuka honey	Chitosan/gelatin cryogel and hydrogel + honey	In vitro	Strong antibacterial properties	Bacterial inhibition, viability, biofilm formation	[97]
Honey	Gelatin + curcumin + honey	In vitro and in vivo (rat model), nanofibrous wound dressing	Enhanced antioxidant and antibacterial activity	DPPH, 2,2′-azino-bis(3-ethylbenzothiazoline-6-sulfonic acid) (ABTS) and ferric reducing antioxidant power (FRAP), bacterial inhibition, wound closure	[89]
Honey	PVA + pomegranate peel extract + honey + bee venom	In vitro and in vivo (rat model), wound healing	Improved healing and antibacterial activity	Antibacterial activity, cytotoxicity, wound closure	[115]
Honey	Chitosan/PVA + honey	In vitro	Potential wound healing application	Physicochemical analysis, bacterial inhibition	[102]
Manuka honey	Polycaprolactone (PCL)/methylcellulose + honey	In vitro	Good cytocompatibility	Cell viability, scratch assay, antibacterial tests	[116]
Honey	Sodium alginate + honey	In vitro and in vivo (rat model), wound healing	Good antibacterial effect	Wound healing, bacterial inhibition	[117]
Propolis	Polyacrylamide (PAM)/methylcellulose + propolis	In vitro	High antioxidant activity	DPPH, ABTS, FRAP, polyphenols	[49]
Propolis	PVA + propolis	In vitro and in vivo (rat model), burn wound healing	Accelerated wound contraction	Wound closure, re-epithelialization	[48]
Propolis	Chitosan/pectin + propolis	In vitro	Improved antibacterial and healing effects	Scratch assay, antibacterial activity	[69]
Propolis	Alginate/chitosan + propolis	In vitro	Controlled release system	Release kinetics, swelling	[99]
Propolis	Poly(lactic-co-glycolic acid) (PLGA)/ hydroxypropyl cellulose (HPC) + propolis	In vitro	Improved antioxidant and cell proliferation	Antibacterial test, antioxidant activity, biocompatibility assays	[118]
Propolis	Carbopol + propolis	In vitro and in vivo (rat model), wound model	Inhibited nitric oxide (NO) production, promising wound healing	NO, prostaglandin E2 (PGE_2_), cytotoxicity, antimicrobial activity, DPPH, wound healing	[119]
Propolis	Bacterial nanocellulose + propolis	In vitro	Reduced *Staphylococcus aureus* growth	Antibacterial assays	[120]
Honey + Propolis	Collagen-based matrix (semi-hydrogel system)	In vitro and in vivo (mouse model), wound healing	Enhanced healing and tissue regeneration	Antioxidant, wound closure, collagen deposition	[92]
(**B**)					
**Bioactive Component**	**Hydrogel Composition**	**Experimental Model/Application**	**Key Outcomes**	**Biomarkers/Parameters Evaluated**	**Ref.**
Propolis	Collagen hydrogel with propolis-loaded ZIF-8 nanoparticles + menstrual blood stem cells	OA treatment model	Local propolis hydrogel delivery in an OA model; further comparator validation needed	OA response, local delivery, hydrogel formulation, comparator validation	[82]
Manuka honey	Gellan gum + inorganic clay + Manuka honey	Cartilage tissue engineering model	Improved mechanical strength; biofilm inhibition in a cartilage-related setting	Biofilm viability, cell differentiation, immunological reaction, antibacterial activity	[121]
ROS-scavenging hydrogel system	In situ forming cartilage matrix-mimetic ROS-scavenging hydrogel	OA after superficial cartilage injury	Localized ROS scavenging; benchmark for OA-specific antioxidant hydrogel design	ROS scavenging, cartilage preservation, OA progression, local hydrogel performance	[24]
Methylprednisolone	Silk fibroin/hyaluronic acid injectable hydrogel + methylprednisolone	Cartilage regeneration model	Injectable local delivery; benchmark for cartilage-relevant drug exposure	Cartilage regeneration, controlled release, biocompatibility, retention/release behavior	[25]
MMP-13 siRNA	ROS-responsive hydrogel with MMP-13 siRNA nanocarriers	OA progression model	ROS-responsive release and suppression of matrix-degrading pathways	MMP-13 targeting, ROS-responsive release, cartilage matrix degradation, OA progression	[60]
Resveratrol	Chondroitin sulfate@resveratrol liposome-loaded ROS-responsive injectable hydrogel	OA alleviation model	ROS-responsive antioxidant delivery and local joint modulation	ROS levels, antioxidant delivery, inflammatory response, cartilage protection	[61]

## Data Availability

No new data were created or analyzed in this study. Data sharing is not applicable to this article.

## Abbreviations

The following abbreviations are used in this manuscript:

OA	Osteoarthritis
ROS	Reactive Oxygen Species
NF-κB	Nuclear Factor Kappa-Light-Chain-Enhancer of Activated B Cells
MAPK	Mitogen-Activated Protein Kinase
Nrf2	Nuclear Factor Erythroid 2-Related Factor 2
HO-1	Heme Oxygenase-1
NADPH	Nicotinamide Adenine Dinucleotide Phosphate
MMPs	Matrix Metalloproteinases
TNF-α	Tumor Necrosis Factor Alpha
IL-1β	Interleukin-1 Beta
IL-6	Interleukin-6
PVA	Polyvinyl Alcohol
RGD	Arginine–Glycine–Aspartic Acid
iNOS	Inducible Nitric Oxide Synthase
COX-2	Cyclooxygenase-2
AP-1	Activator Protein 1
ABTS	2,2′-azino-bis(3-ethylbenzothiazoline-6-sulfonic acid)
FRAP	Ferric reducing antioxidant power
PCL	Polycaprolactone
PAM	Polyacrylamide
PLGA	Poly(lactic-co-glycolic acid)
HPC	Hydroxypropyl cellulose
NO	Nitric oxide
DPPH	2,2-diphenyl-1-picrylhydrazyl
PGE	Prostaglandin E2
ADAMTS	A Disintegrin and Metalloproteinase with Thrombospondin Motifs
SOD	Superoxide Dismutase
GPx	Glutathione Peroxidase
GSH	Glutathione
ECM	Extracellular Matrix
IκBα	Inhibitor of Nuclear Factor Kappa B Alpha
CAT	Catalase
